# Fracture risk, underlying pathophysiology, and bone quality assessment in patients with Turner syndrome

**DOI:** 10.3389/fendo.2022.967857

**Published:** 2022-10-17

**Authors:** Kento Ikegawa, Yukihiro Hasegawa

**Affiliations:** ^1^ Division of Endocrinology and Metabolism, Tokyo Metropolitan Children’s Medical Center, Tokyo, Japan; ^2^ Clinical Research Support Center, Tokyo Metropolitan Children’s Medical Center, Tokyo, Japan; ^3^ Department of Pediatrics, Keio University of School of Medicine, Tokyo, Japan,

**Keywords:** Turner syndrome, fracture, hypogonadism, BMD, QCT, TBS

## Abstract

Turner syndrome (TS), the most common type of X chromosomal disorder, has various, clinical manifestations. Among these, primary hypogonadism, which may lead to osteoporosis, is a life-long health issue. A high prevalence of fractures associated with osteoporosis is a major problem in patients with TS, where it may be 1.4-2.2 times higher than in healthy individuals and increases with age.

Among the risk factors associated with fractures in TS, hypogonadism is arguably the most important. Estrogen deficiency due to hypogonadism leads to low bone mineral density (BMD), resulting in a high prevalence of bone fractures. Estrogen replacement therapy (ERT) in patients with TS reportedly improved their BMD. However, other causes of low BMD may exist, given that this condition begins in the prepubertal period in patients with TS.

Most previous studies have reported low BMD in patients with TS using dual-energy X-ray absorptiometry (DXA), but this method has some limitations. Areal BMD values assessed by DXA were influenced by bone size and short stature, resulting in an underestimation of BMD. Currently, volumetric BMD values may be accurately obtained using peripheral quantitative computed tomography (pQCT). pQCT, high-resolution pQCT, and the trabecular bone score can also be used to evaluate bone quality, including bone geometry and microarchitecture, in TS.

The present review discusses the high fracture risk, role of estrogen deficiency in low BMD, advantages and disadvantages of various bone assessment methods, and characteristics of bone quality in TS.

## 1 Introduction

Turner syndrome (TS) is a congenital disorder resulting from partial or complete loss of one X-chromosome and has a prevalence of approximately one in 2000 live births (1). Patients with TS have various symptoms, such as short stature, hypogonadism, cardiac malformation, and fractures, although their clinical phenotype varies depending on their karyotype (1, 2). A high fracture risk is especially problematic for patients with TS because the prevalence of fractures can be as high as 30.5-32.2% (3–5).

Numerous, previous studies and reviews have reported the characteristics of fractures in TS (2, 3, 6), which are caused by estrogen deficiency, a high risk of falling, X chromosomal abnormalities, and comorbidities of TS (6, 7). In particular low bone mineral density (BMD) stemming from estrogen deficiency is associated with an increased risk of fractures. BMD was higher in patients with TS with a spontaneous menstrual cycle than in those with primary hypogonadism (8–12). Indeed, estrogen replacement therapy (ERT) has been found to improve BMD in TS (13).

Dual-energy X-ray absorptiometry (DXA) is often used to assess BMD in TS but it is crucial to understand its limitations. Areal BMD (aBMD) values obtained using DXA are influenced by bone size and short stature, resulting in an underestimation of BMD (14, 15). This limitation of DXA has implications for patients with TS, most of whom have short stature. In recent years, quantitative computed tomography (QCT) has come to be used more often to assess volumetric BMD (vBMD) (16–19).

Bone quality, including bone geometry and microarchitecture, contribute to increasing or decreasing the risk of fractures independently of BMD (20). Bone quality in TS is currently evaluated using peripheral QCT (pQCT), high-resolution pQCT (HR-pQCT), and the trabecular bone score (TBS).

The present review aims to summarize the high fracture risk, role of estrogen deficiency in low BMD, advantages and disadvantages of various bone assessment methods, and characteristics of bone quality while focusing on TS.

## 2 High fracture risk in Turner syndrome

### 2.1 Epidemiology of the fracture risk in Turner syndrome

Patients with Turner syndrome (TS) have a 30.5-32.2% prevalence of fractures (3–5). Population databases show that the risk of fractures is 1.4-2.2 times higher in patients with TS than in healthy individuals (21, 22). The high fracture risk in these patients has its onset in childhood (21, 23) and increases with age (24, 25). The odds ratio (OR) of the fracture rate between patients with TS and healthy subjects was 1.99 for those younger than age 45 years and 19.26 for those older than 45 years (24). In general, the occurrence of a fracture depends on bone strength and the frequency, type and severity of trauma. Bone strength is determined by bone density and bone quality, including the microarchitecture of cortical and trabecular bone and bone geometry (26). All these factors may contribute to increasing the risk of fractures in patients with TS.

While numerous, previous studies have demonstrated a high fracture risk in patients with TS, some have denied any significant difference from the fracture risk in the general population. Ross et al. compared past fractures in 78 patients with TS aged 4-13 years with the annual fracture rate in healthy children and reported that the total, annual fracture rate did not differ significantly between these groups (27). Another report demonstrated that the total fracture rate in 267 adult patients with TS (30.5%) did not differ significantly from the epidemiological data showing a rate of 32-44% (4). These reports were possibly biased because the data on the fracture rate in patients with TS and healthy controls were collected in different regions and at different times. The impact of the role of ERT on fractures is not entirely clear although its impact on BMD has been studied.

### 2.2 The pathophysiology underlying the high fracture rate in Turner syndrome

A major contributor to fractures in TS is the loss of bone density associated with estrogen deficiency. Estrogen suppresses osteoclasts, and estrogen deficiency weakens this suppression, resulting in osteoclast activation and loss of BMD (28). Estrogen deficiency also contributes to decreased intestinal calcium absorption and increased urinary calcium loss, which occurs when parathyroid hormone secretion decreases in response to elevated serum calcium resulting from bone resorption (29).

Elevated follicle-stimulating hormone (FSH) is also associated with decreased BMD in TS patients (6). FSH decreases bone density by directly binding to FSH receptors on osteoclasts and indirectly by promoting the production of TNF-α, which in turn promotes the production of osteoclasts (30, 31). Sun et al. demonstrated that neither FSHβ nor FSH receptor null mice had decreased BMD despite having severe hypogonadism (30). Furthermore, they demonstrated that BMD increased in haploinsufficient FSHβ^+^ mice with normal ovarian function, suggesting that FSH directly affects bone loss (30). Many patients with TS have a high FSH level from the neonatal period or early childhood (32), and it is possible that a high FSH level is related in some way to decreased BMD.

Bone fragility in TS is caused not only by decreased BMD but also decreased bone quality, including changes in bone geometry and microarchitecture. An increase in the total bone area, low cortical bone thickness in the radius, and small vertebrae and femurs have been reported in patients with TS (17–20, 33) (**Table 1**). The characteristics of radial bone in TS may be caused by a deletion of entire coding regions in the SHOX gene (SHOX-del) (33). The microarchitecture is affected by remodeling, a process of bone formation mediated by osteoblasts, and bone resorption mediated by osteoclasts (7). Remodeling in TS shows an imbalance between these processes, with bone resorption being more pronounced than bone formation (36), resulting in poor trabecular microarchitecture (35).

**Table 1:** Bone density and quality in TS according to QCT, pQCT, and HR-pQCT.

	Age (y)	Number	Method	Area	Cortical/Trabecular	Value of cases (g/cm^3^)	Values of control (g/cm^3^)	Bone Geometry
Nanao et al., 2002 (16)	4-6.9	5	QCT	Vertebral	Total	0.226 (0.036)	0.216 (0.031)	N/A
	7-9.9	8				0.193 (0.029)	0.220 (0.033) ^‡^	
	10-12.9	15				0.177 (0.032)	0.217 (0.017) ^‡^	
Holroyd et al., 2010 (34)	12.7^†^	22	pQCT	Proximal radius	Total	-1.04 (1.06)	-1.29 (1.13)	The cortical thickness in the TS group was reduced
					Cortical	-2.58 (1.30)	-1.38 (1.40) ^‡^	
Soucek et al., 2011 (17)	10.3 (2.2) ^†^	22	pQCT	Proximal radius	Total	0.511 (0.100)	N/A	Cortical area and cortical thickness were low in all age groups.
					Cortical	0.936 (0.059)	N/A	
	14.3 (1.7) ^†^	25			Total	0.653 (0.099)	N/A	
					Cortical	1.026 (0.057)	N/A	
	17.4 (1.2) ^†^	20			Total	0.657 (0.106)	N/A	
					Cortical	1.091 (0.038)	N/A	
Pitukcheewanont et al., 2011 (20)	11.9 (3.3) ^†^	22	QCT	Vertebral	Total	0.230 (0.036)	0.279 (0.048) ^‡^	Vertebrae and femurs of patients with TS were smaller.
				Femoral	Total	2.011 (0.063)	2.033 (0.066)	
Hansen et al., 2012 (35)	35 [20-61]	32	HR-pQCT	Distal radius	Cortical	0.961 [0.83-1.02]	0.922 [0.76-1.00]	Cortical thickness was higher in TS in the radius but not the tibia
					Trabecular	0.112 (0.036)	0.154 (0.044) ^‡^	
				Tibia	Cortical	0.927 [0.81-0.97]	0.890 [0.80-0.96]	
					Trabecular	0.135 (0.040)	0.176 (0.044) ^‡^	
								
								
Soucek et al., 2013 (33)	10.3 (2.2) ^†^	22	pQCT	Distal radius	Trabecular	0.185 (0.033)	0.215 (0.046) ^§^	Increased total bone area and a thin cortex were observed in patients with TS.
Soucek et al., 2015 (18)	15.3 (3.2) ^†^	32	pQCT	Radius	Cortical	1.108 (0.052)	1.137 (0.044) ^‡^	Cortical thickness was misinterpreted as decreased radial bone density due to the partial volume effect.
				Tibia	Cortical	1.104 (0.048)	N/A	
Soucek et al., 2018 (19)	10.0 (2.2) ^†^	15	pQCT	Distal radius	Trabecular	0.186 (0.031)	N/A	Total bone cross-sectional area increased with age.
				Proximal radius	Cortical	1.057 (0.031)	N/A	
	13.5 (1.5) ^†^	14		Distal radius	Trabecular	0.165 (0.031)	N/A	
				Proximal radius	Cortical	1.084 (0.037)	N/A	
	16.1 (0.4) ^†^	3		Distal radius	Trabecular	0.149 (0.015)	N/A	
				Proximal radius	Cortical	1.155 (0.019)	N/A	

y, year; vBMD, volumetric bone mineral density; QCT, quantitative computed tomography; pQCT, peripheral QCT.

^†^Values are expressed as the mean, median or number.

^‡^Statistically significant.

^§^Values of SHOX-D patients.

NA, Not available.

### 2.3 Pathogenetic mechanism of the fracture risk in Turner syndrome

#### 2.3.1 Estrogen deficiency

The most obvious cause of fractures in patients with TS is hypogonadism. ERT reportedly decreased the fracture rate in patients with hypogonadism (14). Patients with primary amenorrhea caused by some other diseases besides TS had a significantly higher fracture prevalence than healthy controls (33% vs 5%) (37), suggesting that hypogonadism was one of the significant clinical risk factors associated with fractures. Gravholt et al. published two manuscripts on the fracture rate in patients with TS, demonstrating the importance of ERT for fracture risk reduction in these patients. In the first study, they used the Danish Population Database to show that the relative risk (95% confidence interval [CI]) of fractures in patients with TS was 2.16 (1.50-3.00) compared to the general population (21). Their second study, which employed a similar method, demonstrated that the hazard ratio (95% CI) for fractures in patients with TS was 1.35 (1.04-1.75) (22). The higher fracture risk found in the first study was the result of a difference in the percentage of subjects receiving ERT between the two studies; in the former study, almost none of the patients had received ERT while in the second study, 83% had, indicating the efficacy of ERT. Although some studies have investigated the relationship between BMD and the timing and dosage of ERT as described below, no reports have hitherto directly investigated the relationship between these ERT-related factors and the frequency of fractures.

#### 2.3.2 High risk of falling

A higher risk of falling associated with hearing impairment also contributes to the high fracture rate in patients with TS. Several, previous studies reported an association between hearing impairment and the fracture rate (4, 5). Hearing impairment leads to impaired speech perception and spatial orientation, increasing the risk of falling resulting in forearm fractures (4). This type of fracture is common in patients with TS, whose annual, childhood incidence of wrist fractures was significantly higher than in healthy children (9.1/1000 vs 3.5/1000, p < 0.003) (27).

#### 2.3.3 X chromosomal abnormalities

Other reasons for the high fracture risk in patients with TS includes X chromosomal abnormalities (7, 38). SHOX-del, which is observed in almost all patients with TS, may be the one of the most important factors. Altered bone shape and microarchitecture, observed in many patients with TS, possibly stem from SHOX-del (39). Children with SHOX deficiency (SHOX-D) had an increased total bone area (Z-score = 1.5 ± 1.4, p = 0.001) and thin cortex (Z-score = -2.0 ± 1.2, p < 0.001) than healthy subjects (33). Similar results were observed in patients with TS (33) (**Table 1**). Although no studies have as of yet examined the higher fracture rate associated with SHOX-D, the previously mentioned differences in bone characteristics may contribute to the higher fracture rate in patients with TS. Other genes associated with fractures in TS include bone morphogenetic protein 2 (*BMP2*), which is involved in bone mineralization; insulin-like growth factor 2 (*IGF2*), which is involved in bone repair and formation; and secreted frizzled-related protein 1 (*sFRP1*), which plays an important role in Wnt signaling (40–42). Genome-wide methylation analysis profiling has shown that these genes are not located on the X chromosome but are downregulated in 45, X cells (43).

#### 2.3.4 Comorbidities of Turner syndrome

Comorbidities of TS, such as obesity, diabetes, inflammatory bowel disease (IBD), and thyroid disorder affect bone health (7, 38). Obesity activates both osteoblast and osteoclast functions (44), and epidemiological data show that obesity increases the fracture risk (45). A systematic review demonstrated that the relative risk (95% CI) of any type of fracture in patients with diabetes mellitus was 1.5 (1.3-1.8) (46). A population study reported an increased fracture risk associated with thyrotoxicosis (47), and a review article reported that patients with IBD had an increased fracture risk (48). However, the nature of the association of thyrotoxicosis and IBD with the fracture rate is still unknown (7).

## 3 Low BMD and estrogen deficiency in patients with Turner syndrome

Patients with TS have low bone mineral density (BMD), which is one of the causes of the high prevalence of fractures (5). An association between BMD and the fracture risk was has been observed both in healthy individuals and patients with TS (5, 49). A cohort study of 124, healthy, subadult females indicated that the fracture risk was significantly higher in healthy subjects with a low total volumetric BMD (vBMD) of the distal radius (odds ratio (OR): 1.71) (49). An interview of 177 adult patients with TS demonstrated that an increased risk of fractures was independently associated with low vertebral BMD as measured by dual-energy X-ray absorptiometry (DXA) (OR: 3.2; 95% CI: 1.0-10.5) (5). Based on these results, BMD is now often used as a surrogate outcome to predict the risk of fractures although other factors, such as bone microarchitecture, bone geometry, and the high risk of trauma owning to hearing impairment described above, are also potential risk factors.

One of the most significant causes of low BMD in patients with TS is estrogen deficiency, a well-established finding of this syndrome, as well as postmenopausal osteoporosis (7, 50, 51). Several facts indicate the importance of estrogen for BMD in patients with TS. First, BMD was higher in patients with TS with a spontaneous menstrual cycle, which occurs in 6% of patients with TS (25, 52), than in those with primary hypogonadism (8–12). Second, the later ERT is initiated, the lower the resulting BMD is (13, 53). Third, the BMD Z-score was lower in adolescence than in early childhood (8), suggesting that BMD did not increase as much during adolescence in these patients as in healthy individuals, who are typically exposed to estrogen. In this study, the mean starting age for ERT was 13.5 (range 7.1-21.3) years, or later than the currently recommended age. Therefore, the BMD Z score might not have decreased in these patients if ERT had been started at the appropriate age.

ERT is known to improve BMD in patients with TS, a finding which was borne out by our own study (54), which compared the BMD Z-score measured by DXA before and after cyclic estrogen and progesterone therapy (Kaufmann therapy) in 18 patients with TS and found an increase in the BMD Z-score after the start of therapy (54). To the best of our knowledge, no other study has compared BMD before and after Kaufmann therapy in the same subjects.

ERT is effective in increasing BMD, but the increase is insufficient and needs to be improved. BMD was higher in patients with TS with a spontaneous menstrual cycle than in those with primary hypogonadism even if they received ERT (8–12), suggesting that there is room for improvement in the conventional ERT. In these reports, the mean starting age of ERT was 13.5-20.2 years, and the mean age of menarche onset was 14.7-17.8 years (8–11). Since BMD is known to increase more with earlier ERT initiation (13), patients with TS may achieve long-term BMD if they begin ERT around the recently recommended age of 11-12 years.

Low BMD in patients with TS reportedly begins before pubertal ages (16, 55), suggesting that hypogonadism might not be the only cause. Nanao et al. measured bone density in the lumbar spine using QCT, which mainly represents trabecular BMD, in 21 subadult females with TS and 20, healthy, age- and sex-matched controls (16). The study demonstrated lower BMD in the patients with TS than in the healthy controls in the prepubertal period (16). The authors also observed a gradual decline in vertebral vBMD before prepubertal age (16) (**Table 1**). Hogler et al. published a study supporting these findings, demonstrating that the decrease in vertebral vBMD in patients with TS occurred before pubertal age (55). One, possible explanation of these findings of low trabecular BMD is the low level of estrogen secretion before pubertal age.

The optimal timing of the start of estrogen replacement therapy for TS is uncertain (10, 54). Saito et al. demonstrated that age at ERT initiation was significantly related to BMD as measured by DXA (53). Another study demonstrated that height, age, and cortical thickness-adjusted cortical vBMD as measured by pQCT was positively correlated to the duration of ERT (17). Recent guidelines recommend that estrogen replacement begin between ages 11 and 12 years and be increased to the adult dosage over 2-3 years (25, 56) although the evidence in terms of BMD is pending.

Furthermore, the optimal, initial dosage and the criteria for increasing the dosage have not been established. One, previous report demonstrated that a high oral 17B-estradiol dosage (4 mg/day) did not affect BMD significantly in adult patients with TS (57). Our previous report demonstrated that BMD in patients with TS who began ethinyl estradiol therapy at an ultra-low dosage (1-5 ng/kg/day) was no different from that of their counterparts receiving classical conjugated estrogen (12). A recent study recommended 3-7 µg/day for transdermal E2 or 0.25 mg/day for 17β oral E2 as the pubertal initiation dose (25) although this recommendation is not evidence-based.

Diet and exercise habits may also contribute to low BMD in patients with TS. The serum vitamin D level was shown to be lower in patients with TS, which may be a contributing cause of lower BMD (38, 58). Physical activity is also reportedly associated with high BMD in patients with TS (59). Thus, an appropriate diet with sufficient vitamin D and exercise are important for bone health, especially in TS (7, 11).

## 4 Bone density and quality assessment methods in patients with Turner syndrome

One of the most common methods of assessing BMD is DXA, a low-dose x-ray technology that measures the attenuation of x-ray beams as they pass through tissues of varying density (15). The recommended measurement sites are the lumbar spine, distal radius, proximal femur, or the whole body for adults, and the lumbar spine or total body less head (TBLH) for children (60, 61). The lumbar spine and TBLH consist mainly of trabecular bone, and the distal radius, proximal femur, and whole body consist primarily of cortical bone (60). DXA is rapid, safe, widely available, and use comparatively lower levels of radiation (15). However, in general, DXA can only measure aBMD, which is expressed in g/cm^2^, but not vBMD, which is expressed in g/cm^3^. This limitation may cause the values to be skewed by short stature and bone size (14, 15). Although there are various methods of using by DXA to assess vBMD, they are generally only theoretical (15). An exception is the method described by Kroger et al., which uses the cylindrical shape of bone to calculate the vBMD using the following formula: vBMD = aBMD*[4/(π *width of vertebral)] (60, 62). This method has been validated for use in children aged 6-19 years (60).

QCT, which is generally a method of evaluating the lumbar spine or proximal femur using computed tomography (CT), allow us to directly measure the vBMD (in g/cm^3^) (15, 63). Therefore, QCT can provide the true BMD value independently of short stature and bone size. Furthermore, this method can be used to evaluate cortical and trabecular bone separately (15, 63). However, one of the disadvantages of QCT is that patients are exposed to high doses of radiation (15, 63).

pQCT is a technique for evaluating radial and tibial BMD using the smaller, less expensive peripheral CT scan (15, 63). This method can assess vBMD using a lower radiation dose (64). HR-pQCT can accurately assess cortical BMD with less partial volume effect (15, 18), and also can measure bone microstructure by using indicators, such as cortical porosity, trabecular number, and trabecular spacing, to produce an assessment of bone quality (65).

TBS is an indirect method of measuring the trabecular microarchitecture based on DXA data (66, 67). TBS is calculated as the sum of the squared gray-level differences between pixels at a specific distance (66, 67). The better the trabecular microarchitecture, the more gray-level variation of small amplitude there is, which leads to increasing the TBS score (67). This method is easy, cost-effective, and involves lower levels of radiation exposure. The disadvantage of TBS is the lack of reference data (66).

### 4.2 Findings and limitations of bone density and quality assessments in Turner syndrome

#### 4.2.1 Findings and limitations of methods of assessing bone density in Turner syndrome

The methods described above are used to assess BMD or bone quality in patients with TS. Numerous, previous studies have diagnosed low BMD in patients with TS using DXA, but this method has limitations, especially when used in patients with TS (7, 14). Bakalov et al. demonstrated the aBMD value on DXA was influenced by bone size and short stature, resulting in an underestimation of BMD in patients with TS (14). They demonstrated that a difference in the BMD values for the femoral neck between patients with TS and age-matched, healthy, adult females decreased after adjusting for bone size (14).

Several reports have assessed the vBMD in patients with TS using either DXA or QCT (16, 38). Gravholt et al. assessed the vBMD of the lumbar vertebrae in patients with TS using DXA (38), which reconstructs the vertebrae using four scans to allow a three-dimensional view. Nanao et al. measured the vBMD of the lumbar vertebrae in patients with TS using QCT, which can directly provide three-dimensional assessment of BMD, and demonstrated that the vBMD had already decreased during the prepubertal period (16). These methods can reduce the distorting effect of bone size and short stature on BMD assessments (15).

Recently, pQCT, which measures radial bone density and involves less radiation exposure than QCT, has come into use (17, 19) (**Table 1**). Several studies used pQCT to show that the cortical BMD in patients with TS during and after puberty was lower than in healthy, adult females (17, 34, 68). However, Soucek et al. demonstrated that the cortical BMD on pQCT did not decrease in patients with TS and that the decline in cortical BMD in TS reported previously was due to the partial volume effect described mentioned above (18), which skews assessments of the cortical BMD, particularly in patients with a cortical thickness < 2.0 mm (63, 69). A previous study using HR-pQCT, which is less affected by the partial volume effect, reported no difference in cortical BMD between patients with TS and control subjects (35).

#### 4.2.2 Findings and limitations of bone quality assessments in Turner syndrome

Bone quality contributes to increased or decreased risk of fractures independently of BMD (26). Recent studies have described bone quality in TS, including bone geometry and microarchitecture.

Bone geometry assessments using QCT and pQCT demonstrated that the total bone area increased, and cortical thickness decreased, in TS (**Table 1**). Soucek et al. demonstrated that the total bone area Z-score (SD) and cortical thickness Z-score (SD) of the proximal radius in prepubertal patients with TS was 0.9 (1.5) and -0.7 (1.2), respectively (33). These findings were more pronounced in patients with SHOX-D (33), suggesting that SHOX-del is a major contributor to altered bone geometry in TS. One of the hypotheses advanced to explain the smaller changes in bone geometry seen in TS than in SHOX-D holds that low serum estrogen might suppress changes in bone geometry (33). Since there are many unknowns regarding the relationship between estrogen and bone geometry, future studies are needed to explore this issue.

Microarchitecture is another important factor determining bone quality and is associated with resistance to fractures (70). A previous study used HR-pQCT to demonstrate weakened trabecular microarchitecture in TS. Hansen et al. compared the radial bone microarchitecture in adult patients with TS and healthy controls using HR-QCT and found that cortical porosity was lower in TS patients (0.58 [0.10-2.27]% versus 1.14 [0.27-2.92]%; p<0.0001) (35). Their study demonstrated a lower trabecular value (1.42 [0.42-2.15] mm^-1^ versus 1.92 [1.77-2.07] mm^-1^; p<0.0001) and higher trabecular spacing (0.65 [0.40-2.28] mm versus 0.44 [0.36-1.9] mm) (35). The TBS, an indirect method of measuring trabecular microarchitecture, is also considered a useful predictor of fractures (71). The TBS is calculated using data from DXA, is less burdensome to patients, and involves less radiation exposure than other methods of evaluating bone microarchitecture, such as HR-pQCT (53, 71). Two, previous studies demonstrated an association between the TBS and fracture history, vertebral and femoral neck BMD, and age in patients with TS (53, 71) (**Table 2**).

**Table 2:** Advantages and disadvantages of bone assessment methods in patients with TS.

	Characteristics	Method	Area	Advantage	Disadvantage
DXA-based	Widely available, low radiation dose	DXA	Vertebrae^†‡^ Distal radius^†^ Proximal femur^†^ Whole body^†^ TBLH^‡^	Common	Influenced by short stature and bone size
		vBMD on DXA	Vertebrae^‡^ Proximal femur^‡^	Three-dimensional assessment	Theorical value
		TBS	Vertebrae^†‡^	Trabecular microarchitecture can be assessed.	Lack of reference data
QCT-based	Three-dimensional assessment, cortical and trabecular BMD can be assessed independently, cortical thickness can be measured	QCT	Vertebrae^†^ Proximal femur^†^	Actual density measurement	High radiation dose
		pQCT	Radius^†‡^	Less radiation exposure than QCT	Affected by partial volume effect
		HR-pQCT	Radius^†‡^	Less affected by partial volume effect	Not clinically available

DXA, dual-energy X-ray absorptiometry; vBMD, volumetric bone mineral density; TBS, trabecular bone score; QCT, quantitative computed tomography; pQCT, peripheral QCT; HR-pQCT, high-resolution pQCT; TBLH, total body less head.

^†^Used for adults.

^‡^Used for children.

Further research is needed to evaluate bone quality, including bone geometry and microarchitecture, and more studies using pQCT, HR-pQCT, and the TBS rather than only DXA are needed to identify the best method and predictor of the fracture risk in patients with TS.

## Author contributions

YH planned the review. KI searched previous studies and wrote the first draft of the manuscript. YH revised the manuscript. All authors contributed to the article and approved the submitted version.

## Funding

YH received a grant from the Japan Agency for Medical Research and Development (AMED 22ek01099464s0403).

## Acknowledgments

We are indebted to Mr. James R. Valera for his assistance with editing this manuscript.

## Conflict of interest

The authors declare that the research was conducted in the absence of any commercial or financial relationships that could be construed as a potential conflict of interest.

## Publisher’s note

All claims expressed in this article are solely those of the authors and do not necessarily represent those of their affiliated organizations, or those of the publisher, the editors and the reviewers. Any product that may be evaluated in this article, or claim that may be made by its manufacturer, is not guaranteed or endorsed by the publisher.
